# Visual–Spatial Ability Predicts Academic Achievement Through Arithmetic and Reading Abilities

**DOI:** 10.3389/fpsyg.2020.591308

**Published:** 2021-04-09

**Authors:** Saifang Liu, Wenjun Wei, Yuan Chen, Peyre Hugo, Jingjing Zhao

**Affiliations:** ^1^School of Psychology, Shaanxi Provincial Key Research Center of Child Mental and Behavioral Health, Shaanxi Normal University, Xi’an, China; ^2^Center for Mental Health Development and Research, Xihua University, Chengdu, China; ^3^Laboratoire de Sciences Cognitives et Psycholinguistique (ENS, EHESS, CNRS), Département d’Etudes Cognitives, Ecole Normale Supérieure, PSL Research University, Paris, France; ^4^INSERM UMRS, Paris Diderot University, Sorbonne Paris Cité, Paris, France; ^5^Department of Child and Adolescent Psychiatry, Robert Debré Hospital, APHP, Paris, France

**Keywords:** visual–spatial ability, reading ability, arithmetic ability, academic achievement, education

## Abstract

This study aimed to investigate how visual–spatial ability predicted academic achievement through arithmetic and reading abilities. Four hundred and ninety-nine Chinese children aged from 10.1 to 11.2 years were recruited and measured visual–spatial, arithmetic, and reading abilities. Their mathematical and Chinese language academic achievements were collected for two consecutive school years, respectively, during the same year as cognitive tests and 1 year after the cognitive tests. Correlation analysis indicated that visual–spatial, arithmetic, and reading abilities and academic achievements were significantly correlated with each other. The structural equation modelling analyses showed that there were two paths from visual–spatial ability to academic achievement: a major path mediated by arithmetic ability and a minor serial mediation path from visual–spatial ability to arithmetic ability to reading ability, then to academic achievement. Results shed light on the importance of visual–spatial ability in education.

## Introduction

It is well documented that academic achievement in primary school significantly impacts children’s physical and mental health (Steinmayr et al., 2015; Bempechat et al., 2018; Bücker et al., 2018; Gremmen et al., 2019). What are the cognitive factors that significantly predict academic achievement in primary school children? How do cognitive factors cooperate to impact academic achievement? Do different domains of academic achievement share common cognitive factors? These questions remain important challenges.

### Role of Visual–Spatial Ability in Learning

Visual–spatial ability as complex cognitive skills involve diverse abilities related to space properties of distance and direction, such as spatial perception, spatial visualization, and mental rotation (Buckley et al., 2018). Recent longitudinal studies demonstrated visual–spatial ability as a key predictor of success in mathematical achievement for primary school children (Gilligan et al., 2017; Geer et al., 2019). Anobile et al. (2017) found that there was an intrinsic link between math and spatial perception. More importantly, visual–spatial ability has been identified to impact general competence of academic achievement in science, technology, engineering, and mathematics (STEM) disciplines in adults eventually (Shea et al., 2001; Wai et al., 2009; Hodgkiss et al., 2018). Visual–spatial ability is no doubt one of the important cognitive components related to academic achievement for school children.

Why is visual–spatial ability important for academic achievement? A dominant hypothesis is obviously that visual–spatial ability is a cognitive precursor directly impacting on academic achievement such as mathematics-related tasks. An alternative assumption might be that visual–spatial ability impacts academic achievement via other cognitive abilities. The aim of the present study was to test these two hypotheses. We examined two cognitive abilities that are possible to be mediators between visual–spatial ability and academic achievement. One was arithmetic ability and the other one was reading ability.

### The Relationship Among Arithmetic Ability, Visual–Spatial Ability, and Academic Achievement

A number of studies have revealed that visual–spatial ability was a predictor of arithmetic ability. Xie et al. (2020) recently performed a meta-analysis of 73 studies and revealed a significant and positive correlation between visual–spatial skills and arithmetic ability [*r* = 0.25, 95% CI (0.21, 0.29), *p* < 0.001]. Agrillo et al. (2003) showed that non-symbolic numerical abilities positively predicted arithmetic achievements measured by addition, subtraction, multiplication, and division. Recent longitudinal studies provided further evidence that visual–spatial ability could predict arithmetic development (Zhang et al., 2014) and arithmetic ability (Lefevre et al., 2010). These results indicated an assumption that visual–spatial ability might impact academic achievement through arithmetic ability.

### The Relationship Among Reading Ability, Visual–Spatial Ability, and Academic Achievement

A number of studies have also reported a link between visual–spatial ability and reading ability (Facoetti et al., 2000; Ek et al., 2009; Franceschini et al., 2012; Boonen et al., 2014; Ruffino et al., 2014; Cui et al., 2019; Liu and Liu, 2019). It has been shown that children with reading disability had deficits in shifting visual–spatial attention (Facoetti et al., 2000; Ruffino et al., 2014). A causal link between visual–spatial attention and reading acquisition was also observed in a longitudinal study (Franceschini et al., 2012). Specifically, Chinese reading needs visual processing and visual perception, which result in that Chinese readers have a high demand on visual skill (McBride-Chang et al., 2005; Siok et al., 2009). Meanwhile, reading ability has been indicated as an important predictor for mathematics achievement for primary school children (Jordon et al., 2002; Grimm, 2008). We therefore speculate that visual–spatial ability might also influence academic achievement via reading ability, especially in Chinese reading.

### Visual–Spatial Ability, Arithmetic Ability, and Reading Ability Cooperate to Impact Academic Achievement

Researchers found that arithmetic and reading ability shared many common cognitive mechanisms. Cui et al. (2019) have showed that arithmetic and reading comprehension have a common fundamental which was visual form perception. A large-scale longitudinal study found that pattern understanding is a predictor of both reading and arithmetic skills (Burgoyne et al., 2019). Furthermore, reading and mathematical skills shared common cognitive skills such as rapid automatized naming (Hecht et al., 2001; Georgiou et al., 2013; Koponen et al., 2016) and inhibition (Koponen et al., 2007; Korpipää et al., 2017; Balhinez and Shaul, 2019; Child et al., 2019).

We have speculated that visual–spatial ability may affect students’ academic achievement through arithmetic or reading ability. Also, there is a positive link between arithmetic and reading ability. Therefore, we suspect that these three factors may have a complex interaction to influence academic achievement. However, it is not clear how visual–spatial ability, arithmetic ability, and reading ability cooperate to impact academic achievement, which will be one of the questions to be investigated in this study.

### The Current Study

In sum, the primary aim of the current study was to explore how visual–spatial ability impacts children’s academic achievement. Specifically, we investigated whether there was a direct impact from visual–spatial ability to academic achievement or alternatively visual–spatial ability influenced academic achievement via arithmetic and reading abilities. The second aim of the present study was to examine whether the impact of visual–spatial ability on academic achievement was domain specific for mathematics only or domain general for both mathematics and language.

## Materials and Methods

### Participants

Four hundred and ninety-nine participants (256 boys, 243 girls) aged from 112 to 133 months (age *M* = 120.77, *SD* = 3.48) in a suburban primary school of Xi’an (10 classes altogether) participated in the study. A total of 499 students participated, yet only 490 of them fully finished the whole tests (seven students were absent from the fifth-grade academic achievement test, and two students transferred to other schools). All children were native Chinese speakers, and have normal vision and hearing abilities. The study was consented by all the participants, their parents, and teachers.

### Measurements

#### Fifth-Grade Measurements

As verbal cognitive factors, such as rapid automatized naming (Georgiou et al., 2013; Koponen et al., 2016), phonological awareness (Su et al., 2018), and morphological awareness (McBride-Chang et al., 2005; Liu et al., 2012), and non-verbal IQ are known to be factors strongly impact reading ability, these factors were also measured and controlled in this study. In the fall semester of the fifth grade (T1), visual–spatial ability, arithmetic ability, and non-verbal IQ were tested in groups. Reading ability and reading-related cognitive tests (rapid automatized naming, phonological awareness, and morphological awareness) were individually administered to each child. Each child completed all tests with about 3.5 h in total.

Visual–spatial subset (MT2) in Chinese Rating Scale of Pupil’s Mathematics Abilities (C-RSPMA) established by Tongji Medical College was used to measure each child’s *visual–spatial ability* (Wu and Li, 2006). C-RSPMA was based on the HRT 1–4 established by Heidelberg University (Haffner, 2005). MT2 consists of five tests including number continuation (3 min), visual length (3 min), figure counting (1 min), square counting (3 min), and number connection (2 min). Each child was asked to write down as many answers as accurately and quickly as possible in the given amount of time for each test. One point is counted for a correct answer within the time limit in these tasks.

Arithmetic subset (MT1) in C-RSPMA was used to measure each child’s *arithmetic ability*. MT1 consists of six tests including addition (1 min), subtraction (1 min), multiplication (1 min), division (1 min), comparison (1 min), and blank filling (2 min). The test format of MT1 was the same as MT2.

Character recognition task was used to measure *reading ability* (Liu et al., 2012; Su et al., 2018). Each participant was asked to read aloud a list of 150 Chinese characters taken from primary-school reading textbooks. These 150 characters were divided into 10 pages. The characters in the test were listed in increasing level of difficulty. Children were asked to read the list from the beginning and complete the entire list. Number of correctly named characters was counted as the measurement of reading ability of each child.

*Rapid automatized naming* (RAN) was assessed using digit rapid automatized naming task (Shu et al., 2006; Lei et al., 2011; Pan et al., 2011). In this task, five digits (7, 4, 6, 9, 3) were repeated eight times on a single sheet of paper and presented in random order from top to bottom on the sheet. Individuals were asked to read aloud the digits in order as accurately and quickly as possible two times. The average naming latencies of the two times was considered as the final score.

*Phonological awareness* was measured by *onset and rime deletion* and *phoneme deletion*. In the task of *onset and rime deletion*, the experimenter orally presented a one-syllable word. The child was asked to remove the onset or rime of the syllable and say the rest of the syllable. For example, the “Lang2” without “L” would be “ang2.” This task consists of 16 items. In the task of *phoneme deletion*, the experimenter orally presented a one-syllable word. The child was asked to remove a given phoneme of the syllable and say the rest of the syllable. For example, the “shuang3” without “u” would be “shang3.” This task consists of 16 items (Su et al., 2018).

Morphological production task was used to measure *morphological awareness* of each child (Shu et al., 2006; Song et al., 2015). Two-morpheme words were orally shown to each subject. One of the two words is the target morpheme, for example, the target morpheme/bao1/(food) from/(bread). Subjects were asked to produce two new words. The new words must include the target morpheme, and one corresponds to the target morpheme (/bao1 zi0/, food) and the other does not (/qian2 bao1/, wallet). This task consists of 16 items.

Raven’s Progressive Matrices were used to assess *non-verbal IQ* of each participant. This test has been widely used in many reading and math studies (e.g., Burgoyne et al., 2019). In this task, subjects were asked to select one of six pieces to complete the puzzle picture with a piece missing.

*Academic achievement* of each child was estimated by their major curriculum tests of the school year evaluations in their fifth grade, including curriculum examination scores of mathematics and Chinese. The examination scores in the fifth grade were collected in the fall semester of the fifth grade (T1). The average score of the mid-term and final mathematical/Chinese curriculum tests in the fall semester of the fifth grade (MT5/CT5) was used as an index of each child’s concurrent mathematical/Chinese achievement.

#### Sixth-Grade Measurements

The examination scores in the sixth grade were collected in the fall semester of the sixth grade (T2). The average score of the mid-term and final mathematical/Chinese curriculum tests in the fall semester of the sixth grade (MT6/CT6) was used as an index of each child’s longitudinal mathematical/Chinese achievement.

### Data Analysis

A correlational analysis of all measures was first performed. Then, hierarchical regression analysis and path model analysis were further conducted to explore mediation effect of arithmetic ability between visual–spatial ability and reading ability. In hierarchical regression analysis of reading ability, non-verbal IQ and reading-related cognitive measures were entered into the model in the first step (step 1); then measures of arithmetic ability or visual–spatial ability were entered into the model in the second step (step 2a or step 2b); finally, all the remaining measures were entered into the model (step 3). In the path model analysis (model 1), reading ability was defined as a dependent variable, with visual–spatial ability as an independent variable, arithmetic ability as a mediating variable, and reading-related cognitive factor and non-verbal IQ were defined as covariates.

Finally, three path models were conducted to investigate whether the prediction from visual–spatial ability to academic achievement was through arithmetic ability and/or reading ability. In the first two path models (models 2A and 2B), academic achievement of the fifth grade and the sixth grade was put into the model as dependent variable separately to examine the concurrent and longitudinal mediation effects, respectively. Visual–spatial ability was entered into the model as independent variable, with arithmetic and reading abilities as mediators and non-verbal IQ as a covariate. In the last path model (model 3), a new latent variable academic achievement was defined as dependent variable by combing Chinese and mathematical curriculum tests of the fifth and sixth grades. The other variables of model 3 were the same as model 2.

## Results

### Descriptive Statistics and Correlational Analysis

Descriptive statistics including means, SDs, minimum scores, and maximum scores of all measures are shown in Table 1. Correlations between all measures are shown in Table 2. Significant correlations (survived Bonferroni correction, *p* < 0.00024) are indicated with a star in Table 2. Almost all correlations between visual–spatial tests and arithmetic, reading, and academic achievement tests were significant.

**TABLE 1:** Descriptive statistic of all measures.

	N	Mean	*SD*	Min	Max	Skewness	Kurtosis
Gender (male/female)	499	256/243					
Age/months	488	120.77	3.48	112	133	0.16	−0.55
**Cognitive variables**							
Non-verbal IQ (/60)	499	45.88	5.72	21	59	−0.77	0.97
RAN (/s)	499	13.86	2.85	8	25	0.79	0.81
Onset and rime deletion (/16)	499	14.23	2.16	6	16	−1.26	0.90
Phoneme deletion (/16)	499	12.30	3.01	0	16	−0.97	0.72
Morphological production (/30)	499	21.79	4.19	9	30	−0.51	0.07
**Reading ability**							
Character recognition (/150)	499	120.70	12.41	69	143	−1.16	1.72
**Arithmetic ability**							
Addition (/40)	499	26.23	4.17	10	37	−0.27	0.03
Subtraction (/40)	499	25.63	4.56	14	37	−0.13	−0.47
Multiplication (/40)	498	30.27	3.16	17	39	−1.14	2.13
Division (/40)	499	26.47	6.40	9	40	−0.39	−0.57
Blank filling (/40)	499	26.66	5.44	7	40	−0.57	0.72
Comparison (/40)	499	25.81	4.62	11	40	−0.26	0.49
**Visual–spatial ability**							
Number continuation (/20)	499	15.26	1.84	10	20	−0.07	−0.22
Visual length (/24)	499	14.70	5.26	0	24	−0.83	−0.19
Figure counting (/21)	499	12.97	3.22	3	21	0.11	−0.00
Square counting (/28)	499	19.50	4.00	3	27	−0.67	0.37
Number connection (/200)	499	96.66	21.89	22	165	−0.13	0.35
**Academic achievement**							
5th grade Chinese curriculum test (/100)	491	83.03	8.84	34	97	−1.77	5.70
5th grade Math curriculum test (/100)	492	87.58	8.54	37	100	−1.81	5.17
6th grade Chinese curriculum test (/100)	490	88.64	6.93	50	98	−2.09	6.55
6th grade Math curriculum test (/100)	490	84.65	12.67	20	100	−1.88	5.02

**TABLE 2:** Correlations of all measures.

	CT6	MT6	CT5	MT5	RA	C1	C2	C3	C4	C5	C6	V1	V2	V3	V4	V5	X1	X2	X3	X4	X5
CT6	1																				
MT6	0.73*	1																			
CT5	0.78*	0.63*	1																		
MT5	0.66*	0.79*	0.71*	1																	
RA	0.56*	0.44*	0.57*	0.45*	1																
C1	0.42*	0.53*	0.44*	0.53*	0.43*	1															
C2	0.43*	0.56*	0.49*	0.57*	0.45*	0.77*	1														
C3	0.41*	0.47*	0.45*	0.47*	0.43*	0.66*	0.62*	1													
C4	0.46*	0.55*	0.49*	0.55*	0.49*	0.69*	0.75*	0.69*	1												
C5	0.53*	0.64*	0.54*	0.64*	0.46*	0.73*	0.77*	0.59*	0.70*	1											
C6	0.37*	0.44*	0.39*	0.47*	0.40*	0.61*	0.61*	0.52*	0.59*	0.66*	1										
V1	0.30*	0.37*	0.27*	0.36*	0.33*	0.45*	0.49*	0.32*	0.46*	0.50*	0.38*	1									
V2	0.28*	0.40*	0.27*	0.42*	0.21*	0.32*	0.33*	0.15	0.27*	0.36*	0.28*	0.20*	1								
V3	0.35*	0.39*	0.36*	0.39*	0.35*	0.55*	0.56*	0.42*	0.48*	0.58*	0.53*	0.37*	0.34*	1							
V4	0.23*	0.30*	0.18*	0.31*	0.17	0.34*	0.34*	0.19*	0.23*	0.40*	0.30*	0.29*	0.42*	0.44*	1						
V5	0.20*	0.23*	0.24*	0.19*	0.24*	0.32*	0.27*	0.30*	0.25*	0.32*	0.35*	0.25*	0.21*	0.33*	0.20*	1					
X1	0.46*	0.49*	0.42*	0.47*	0.33*	0.38*	0.36*	0.26*	0.34*	0.46*	0.34*	0.34*	0.36*	0.33*	0.41*	0.20*	1				
X2	−0.36*	−0.30*	−0.36*	−0.30*	−0.42*	−0.45*	−0.38*	−0.52*	−0.43*	−0.37*	−0.39*	−0.22*	−0.11	−0.32*	−0.11	−0.31*	−0.21*	1			
X3	0.26*	0.24*	0.26*	0.23*	0.23*	0.14	0.19*	0.19*	0.19*	0.20*	0.14	0.10	0.16	0.15	0.11	0.18*	0.20*	−0.17	1		
X4	0.43*	0.40*	0.43*	0.38*	0.33*	0.26*	0.26*	0.27*	0.32*	0.32*	0.21*	0.17	0.20*	0.23*	0.14	0.20*	0.26*	−0.29*	0.54*	1	
X5	0.34*	0.27*	0.33*	0.27*	0.48*	0.26*	0.32*	0.26*	0.29*	0.34*	0.23*	0.25*	0.15	0.23*	0.12	0.13	0.31*	−0.27*	0.21*	0.36*	1

*p < 0.00024 with Bonferroni-correction. C/MT6: Chinese/math curriculum test in sixth grade; C/MT5: Chinese/math curriculum test in fifth grade. RA: reading ability. C1–C6: addition; subtraction; multiplication; division; comparison; blank filling. V1–V5: number continuation; visual length, figure counting, square counting; number connection. X1: IQ; X2: RAN; X3: onset and rime deletion; X4: phoneme deletion; X5: morphological production.

### Visual–Spatial, Arithmetic, and Reading Abilities

**FIGURE 1:**
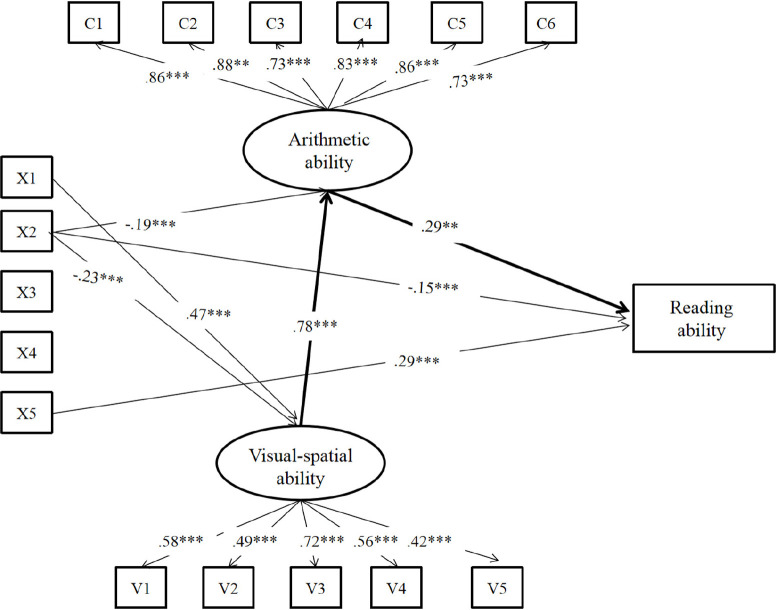
Mediation effect of arithmetic ability between visual–spatial ability and reading ability. V1–V5: number continuation; visual length; figure counting; square counting; number connection. C1–C6: addition; subtraction; multiplication; division; comparison; blank filling. X1: IQ; X2: RAN; X3: onset and rime deletion; X4: phoneme deletion; X5: morphological production. One-headed arrows represent significant paths; non-significant paths are not represented. *^∗∗^p* < 0.01, *^∗∗∗^p* < 0.001.

Results from hierarchical regression analyses (see Table 3) showed that there was significant difference between step 2 and step 1 (step 2a: Δ*R*^2^ = 0.061, *p* < 0.001; step 2b: Δ*R*^2^ = 0.033, *p* < 0.001). Importantly, there was significant difference between step 3 and step 2b (Δ*R*^2^ = 0.033, *p* < 0.001), while no difference between step 3 and step 2a was found (Δ*R*^2^ = 0.005, *p* > 0.05). Results suggest that contribution of visual–spatial ability to reading ability was mediated by arithmetic ability, which was further confirmed by a path model analysis. The model as shown in Figure 1 was a good fit for the data, *χ*^2^*/df* = 3.56, *p* < 0.001, *RMSEA* = 0.07, *CFI* = 0.92, *TLI* = 0.90, and *SRMR* = 0.05, suggesting visual–spatial ability did not contribute to reading ability directly but via arithmetic ability.

**TABLE 3:** Hierarchical regression models of reading ability predicted by visual–spatial ability and arithmetic ability.

Predictors	Reading ability			
	*Step 1 B (SE)*	*Step 2a B (SE)*	*Step 2b B (SE)*	*Step 3 B (SE)*
**Cognitive variables**				
Non-verbal IQ	0.31 (0.09)***	0.14 (0.09)	0.18 (0.09)*	0.13 (0.09)
RAN	−1.16 (0.17)***	−0.67 (0.19)***	−0.95 (0.18)***	−0.65 (0.19)***
Onset and rime deletion	0.30 (0.25)	0.30 (0.24)	0.29 (0.25)	0.29 (0.24)
Phoneme deletion	0.28 (0.19)	0.13 (0.19)	0.22 (0.19)	0.13 (0.19)
Morphological production	0.97 (0.12)***	0.88 (0.12)***	0.89 (0.12)***	0.86 (0.12)***
**Arithmatic ability**				
Addition		0.03 (0.19)		−0.01 (0.19)
Subtraction		0.07 (0.18)		0.03 (0.19)
Multiplication		0.13 (0.21)		0.15 (0.22)
Division		0.35 (0.12)*		0.33 (0.12)**
Comparison		0.07 (0.15)		0.04 (0.15)
Blank filling		0.18 (0.13)		0.15 (0.14)
**Visual–spatial ability**				
Number continuation			0.82 (0.27)**	0.39 (0.29)
Visual length			0.08 (0.10)	0.04 (0.10)
Figure counting			0.47 (0.17)**	0.17 (0.18)
Square counting			−0.14 (0.14)	−0.12 (0.13)
Number connection			0.02 (0.02)	0.01 (0.02)
	*R^2^* = 0.347	*R^2^* = 0.408	*R^2^* = 0.380	*R^2^* = 0.413

*p < 0.05, **p < 0.01, ***p < *0.001.*

### Visual–Spatial Ability and Academic Achievement

**FIGURE 2:**
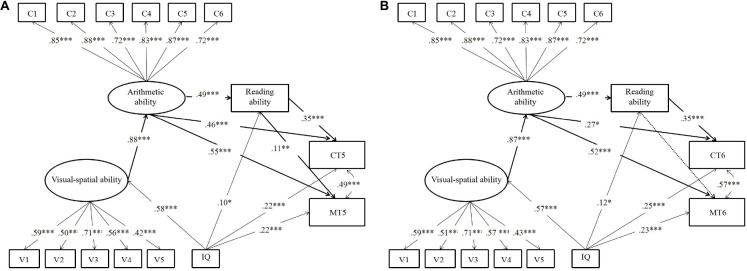
Mediation effects of arithmetic and reading abilities between visual–spatial ability and academic achievement concurrently **(A)** and longitudinally **(B)**. V1–V5: number continuation; visual length; figure counting; square counting; number connection. C1–C6: addition; subtraction; multiplication; division; comparison; blank filling. MT5: math curriculum test in the fifth grade; CT5: Chinese curriculum test in the fifth grade. MT6: math curriculum test in the sixth grade; CT6: Chinese curriculum test in the sixth grade. Twin-headed arrows represent correlations between variables. One-headed arrows represent significant paths; non-significant paths are not represented. *^∗^p* < 0.05, *^∗∗^p* < 0.01, *^∗∗∗^p* < 0.001.

Results of path model analyses as shown in Figure 2 illustrates the mediation effect of arithmetic and reading abilities between visual–spatial ability and academic achievement both concurrently (model 2A: Figure 2A) and longitudinally (model 2B: Figure 2B). Both models were good fit for the data (model 2A:

*χ*^2^/*df* = 3.90, *RMSEA* = 0.08, *CFI* = 0.93, *TLI* = 0.91, and *SRMR* = 0.05; model 2B: *χ*^2^/*df* = 3.58, *RMSEA* = 0.07, *CFI* = 0.94, *TLI* = 0.92, and *SRMR* = 0.05). As shown in model 2A, the paths from visual–spatial ability to arithmetic ability (β = 0.88, *p* < 0.001), arithmetic ability to reading ability (β = 0.49, *p* < 0.001), arithmetic ability to CT5 (β = 0.46, *p* < 0.001), arithmetic ability to MT5 (β = 0.55, *p* < 0.001), reading ability to CT5 (β = 0.35, *p* < 0.001), and reading ability to MT5 (β = 0.11, *p* < 0.05) were significant. However, the paths from visual–spatial ability to CT5 (β = –0.21, *p* = 0.107) and MT5 (β = –0.04, *p* = 0.738) were not significant. As shown in model 2B, the paths from visual–spatial ability to arithmetic ability (β = 0.87, *p* < 0.001), arithmetic ability to reading ability (β = 0.49, *p* < 0.001), arithmetic ability to CT6 (β = 0.27, *p* < 0.05), arithmetic ability to MT6 (β = 0.52, *p* < 0.001), and reading ability to CT6 (β = 0.35, *p* < 0.001) were significant too. However, the path from reading ability to MT6 was marginally significant (β = 0.09, *p* = 0.068), and the path from visual–spatial ability to CT6 (β = –0.05, *p* = 0.680), and MT6 (β = –0.02, *p* = 0.853) was not significant. Model 2A and model 2B demonstrated a similar pattern that there was no direct impact from visual–spatial ability to academic achievement. Model 2A and model 2B also showed two similar types of mediation effects: (1) a major mediation path from visual–spatial ability to arithmetic ability to math or Chinese curriculum test, and (2) a minor serial mediation path from visual–spatial ability to arithmetic ability to reading ability, then to math or Chinese curriculum test. The only difference between model 2A and model 2B was that the mediation path from visual–spatial ability to arithmetic ability to reading ability, then to math performance was significant in model 2A but became marginally significant in model 2B. Table 4 shows B values and proportions of all indirect effects for model 2A and model 2B. Results indicate a similar pattern between concurrent and longitudinal academic achievement, as well as between academic achievement of mathematics and language.

**TABLE 4:** Indirect effects from visual–spatial ability to academic achievement.

	**Paths**	**Indirect effect (*B*)**	***p***
**Model 2A**	**Visual–spatial ability** → **5th grade math curriculum test**		
	Visual–spatial ability→ arithmetic ability→ math curriculum test	0.48 (90%)	< 0.0001
	Visual–spatial ability→ arithmetic ability→ reading ability→ math curriculum test	0.05 (10%)	= 0.0110
	**Visual–spatial ability**→ **5th grade Chinese curriculum test**		
	Visual–spatial ability→ arithmetic ability→ Chinese curriculum test	0.41 (73%)	< 0.0001
	VSL ability→arithmetic ability→ reading ability→ Chinese curriculum test	0.15 (27%)	< 0.0001
**Model 2B**	**Visual–spatial ability**→ **6th grade math curriculum test**		
	Visual–spatial ability→ arithmetic ability→ math curriculum test	0.46 (94%)	< 0.0001
	Visual–spatial ability→ arithmetic ability→ reading ability math curriculum test	0.04 (6%)	= 0.078
	**Visual–spatial ability**→ **6th grade Chinese curriculum test**		
	Visual–spatial ability→ arithmetic ability→ Chinese curriculum test	0.24 (62%)	= 0.018
	Visual–spatial ability→ arithmetic ability→ reading ability→ Chinese curriculum test	0.15 (38%)	< 0.0001
**Model 3**	**Visual–spatial ability**→ **Academic achievement**		
	Visual–spatial ability→ arithmetic ability→ academic achievement	0.50 (83%)	< 0.0001
	Visual–spatial ability→ arithmetic ability→ reading ability→ academic achievement	0.10 (17%)	< 0.0001

**FIGURE 3:**
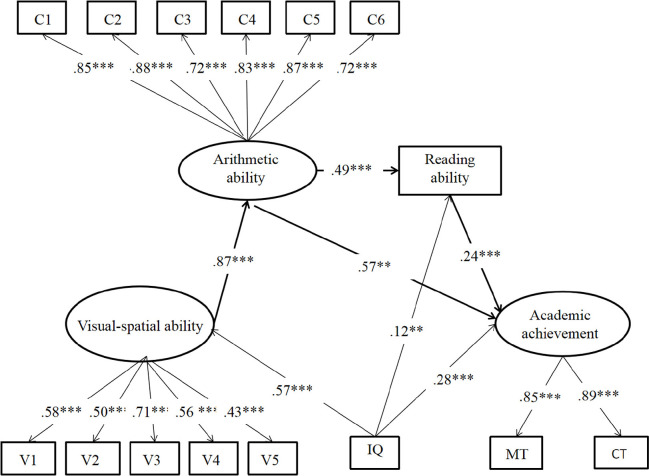
Mediation effects of arithmetic and reading abilities between visual–spatial ability and academic achievement. V1–V5: number continuation; visual length; figure counting; square counting; number connection. C1–C6: addition; subtraction; multiplication; division; comparison; blank filling. MT: average scores on math curriculum test in the fifth and sixth; CT: average scores on Chinese curriculum test in the fifth and sixth. One-headed arrows represent significant paths; non-significant paths are not represented. *^∗∗^p* < 0.01, *^∗∗∗^p* < 0.001.

The final path model (model 3) as shown in Figure 3 was also not surprisingly a good fit for the data, *χ*^2^/*df* = 4.33, *RMSEA* = 0.08, *CFI* = 0.92, *TLI* = 0.90, and *SRMR* = 0.05. As shown in model 3, the paths from visual–spatial ability to arithmetic ability (β = 0.87, *p* < 0.001), arithmetic ability to reading ability (β = 0.49, *p* < 0.001), arithmetic ability to academic achievement (β = 0.57, *p* < 0.01), and reading ability to academic achievement (β = 0.24, *p* < 0.001) were significant. The path from visual–spatial ability to academic achievement was not significant (β = –0.02, *p* = 0.853). Model 3 indicated no direct impact from visual–spatial ability to general academic achievement. Two mediation paths showed significant effects: (1) a major mediation path from visual–spatial ability to arithmetic ability to general academic achievement accounting 83% of the total indirect effects, and (2) a minor serial mediation path from visual–spatial ability to arithmetic ability to reading ability, then to general academic achievement accounting for 17% of the total indirect effects (Table 4).

## Discussion

In the present study, we sought out to determine how visual–spatial ability influenced academic achievement. We found that the relationship between children’s visual–spatial ability and academic achievement was mediated by arithmetic ability and reading ability. In detail, there were two indirect paths from children’s visual–spatial ability to academic achievement. One pathway showed that visual–spatial ability indirectly influenced academic achievement via arithmetic ability (visual–spatial ability → arithmetic ability → academic achievement). The other pathway suggested that arithmetic ability and reading ability played a chain mediating role between visual–spatial ability and academic achievement (visual–spatial ability → arithmetic ability → reading ability → academic achievement).

The most important finding of the present study might be that arithmetic ability played a major role in mediating the impact from visual–spatial ability to academic achievement. Our results showed that the mediation effect solely by arithmetic ability accounted for 83% of the total effects and the remaining 17% serial mediation effect was also partly operated by arithmetic ability. These results enlighten that the impact from visual–spatial ability to academic achievement was mainly driven by arithmetic ability. This might be due to a strong association between visual–spatial ability and arithmetic ability in our dataset, which was consistent with previous well-established findings (see review and meta-analysis by Xie et al., 2020). More importantly, the mediation effect by the arithmetic ability was too strong to overwhelm direct effects from visual–spatial ability to academic achievement. We defer consideration of explanations of the absence of direct effect and its implication for future research later in the discussion.

Second, reading ability also played a role in mediating the impact from visual–spatial ability to academic achievement, but the role was relatively minor and fully dependent on its link with arithmetic ability. The serial mediation effects by arithmetic and reading abilities highlighted the close relationship between the two mediators. Indeed, common foundations between reading and arithmetic abilities have been identified by cognitive approaches (Korpipää et al., 2017; Cui et al., 2019), behavioral genetic methods (Child et al., 2019), longitudinal data (Durand et al., 2005), and neurobiological evidence (Dehaene et al., 2004). However, it should be noted that the serial mediation effects were weak, especially for math curriculum test. This might be caused by weak associations between math curriculum tests and reading ability measured by character recognition task in this study. Reading comprehension task might be a better predictor than character recognition for math curriculum tests in higher grades of primary school as they involve more of word-solving problems. We leave this hypothesis for a test in future research.

Last but not least, similar mediation effects were observed for academic achievement of mathematics and language. This implies a domain-general perspective for common underlying cognitive predictors and similar information processing pathways shared by mathematical and language achievement. This commonality might be due to high correlations between mathematical and Chinese test scores in our dataset that was similar to the findings reported by Spearman (1904). Nevertheless, we are reluctant to accept the notion Spearman proposed, that is, general intelligence explain a large share of variance in all tests, because we have ruled out the influence of non-verbal IQ in our model by adding it as a covariate. We rather see our findings reflect visual–spatial ability as a shared cognitive root among various domains of academic achievement that might not be limited to mathematics and language, but broader to other disciplines of STEM or even social sciences.

Finally, it is worth noting the counterintuitive fact that direct impacts from visual–spatial ability to reading ability and academic achievement were absent in our results. A possible explanation was that the task that we used to measure visual–spatial ability also required counting sequence knowledge that was found closely related to both spatial ability and arithmetic development and played a mediation role between them (Zhang et al., 2014). Therefore, our visual–spatial task with more quantitative characteristics might hinder to observe direct impacts to reading ability and academic achievement but enlarge indirect impacts from visual–spatial ability to arithmetic ability to reading ability and academic achievement. Previous studies have demonstrated that different sub-domains of spatial skills related to mathematical skills in a task- and age-dependent manner (Gilligan et al., 2019). Given the data in hand, we cannot rule out the possibility that other visual–spatial tasks (e.g., Franceschini et al., 2012; also see Newcombe and Shipley, 2015) or elementary school children in different age ranges than our study would provide a strong test of the direct effect. These hypotheses are open to be tested in future studies.

## Limitations, Future Directions, and Implications

It is appropriate to mention at least two limitations of this study. One is that parental socioeconomic level, which may play a role in interrelationships between visual–spatial ability, arithmetic and reading abilities, and academic achievement, was not included in this study. A previous study has proved that as a result of socioeconomic disparities, there is a difference of language experience in children, which in turn leads to differences in brain structure and reading skills (Merz et al., 2020). Some studies also suggest that parental socioeconomic levels play a role in students’ academic achievement (e.g., Tesfagiorgis et al., 2020). Another is that there are multiple dimensions in visual–spatial ability and different dimensions of visual–spatial ability are related to different cognitive abilities (Newcombe and Shipley, 2015). In this study, we did not discuss in detail whether each dimension of visual–spatial ability has the same influence on academic achievement.

In future studies, we should pay more attention to the following: First, as this paper has mentioned, parental socioeconomic levels would be possible to play a role in the influence mechanism of visual–spatial ability on children’s academic achievements. Thus, it would be valuable to explore the interaction between socioeconomic level and visual–spatial ability on children’s academic achievement. Second, some studies have proved that through the training of basic cognitive abilities, an individual’s mathematical ability and reading ability could be improved significantly (Chein and Morrison, 2010; Karbach et al., 2015). Therefore, based on the findings of this study, intervention studies could be conducted to explore whether subjects’ academic achievement can be significantly improved by giving children some training in visual–spatial ability.

In conclusion, the present literature furnishes strong evidence of a longitudinal relationship between visual–spatial ability and academic achievement in Chinese students. Moreover, the findings underscore the potential implication of visual–spatial ability to improve children’s academic achievement. The findings have crucial theoretical implications, suggesting that arithmetic and reading abilities are essential to understand the paths from visual–spatial ability to academic achievement.

On a practical front, according to our model, the worse a child’s starting level of visual–spatial ability, the worse academic achievement they have for older. Thus, researchers can try to measure the visual–spatial ability of the first-grade students so as to predict the academic achievement of the children in primary school.

## Conclusion

The most salient finding of this study pertains to the theoretical model for the impact from visual–spatial ability to the academic achievement in elementary school students. Our study suggests that visual–spatial ability has an indirect impact on primary students’ academic achievement mediated by arithmetic ability and reading ability.

## Data Availability Statement

The original contributions presented in the study are included in the article/supplementary material, further inquiries can be directed to the corresponding author.

## Ethics Statement

The studies involving human participants were reviewed and approved by Shaanxi Normal University. Written informed consent to participate in this study was provided by the participants’ legal guardian/next of kin.

## Author Contributions

SL processed the data and wrote the manuscript. YC and WW collected the data. PH designed the work. JZ designed the work and wrote the manuscript.

## Conflict of Interest

The authors declare that the research was conducted in the absence of any commercial or financial relationships that could be construed as a potential conflict of interest.
